# Pressure-induced topological phases of KNa_2_Bi

**DOI:** 10.1038/srep24137

**Published:** 2016-04-11

**Authors:** I. Yu. Sklyadneva, I. P. Rusinov, R. Heid, K.-P. Bohnen, P. M. Echenique, E. V. Chulkov

**Affiliations:** 1 Donostia International Physics Center (DIPC), 20018 San Sebastián/Donostia, Basque Country, Spain; 2 Karlsruher Institut für Technologie, Institut für Festkörperphysik, D-76021 Karlsruhe, Germany; 3 Institute of Strength Physics and Materials Science, pr. Academicheskii 2/1, 634021, Tomsk, Russian Federation; 4 Tomsk State University, 634050, Tomsk, Russian Federation; 5 St. Petersburg State University, 199034, St. Petersburg, Russian Federation; 6 Departamento de Física de Materiales, Facultad de Ciencias Químicas, UPV/EHU, Apdo. 1072, 20080 San Sebastián/Donostia, Basque Country, Spain; 7 Centro de Física de Materiales CFM - Materials Physics Center MPC, Centro Mixto CSIC-UPV/EHU, 20018 San Sebastian/Donostia, Spain

## Abstract

We report an *ab initio* study of the effect of hydrostatic pressure and uniaxial strain on electronic properties of KNa_2_Bi, a cubic bialkali bismuthide. It is found that this zero-gap semimetal with an inverted band structure at the Brillouin zone center can be driven into various topological phases under proper external pressure. We show that upon hydrostatic compression KNa_2_Bi turns into a trivial semiconductor with a conical Dirac-type dispersion of electronic bands at the point of the topological transition while the breaking of cubic symmetry by applying a uniaxial strain converts the compound into a topological insulator or into a three-dimensional Dirac semimetal with nontrivial surface Fermi arcs depending on the sign of strain. The calculated phonon dispersions show that KNa_2_Bi is dynamically stable both in the cubic structure (at any considered pressures) and in the tetragonal phase (under uniaxial strain).

Alkali metal bismuthides form a group of compounds which attract much attention as materials with a variety of interesting physical properties. The Bi-based binary compounds, A_3_Bi (A = Na, K, Rb), belong to a special class of topological electronic states, three-dimensional (3D) Dirac semimetals^1^,^2^,^3^, where the valence and conduction bands touch at discrete points at the Fermi level. Such materials with a conical 3D dispersion of electronic bands at one or more crossing points and with nontrivial surface states (so-called Fermi arcs) are being given much attention^1^,^2^,^3^,^4^,^5^,^6^,^7^,^8^.

The 3D conical dispersion relations can also be achieved at the point of semimetal (metal) - to - semiconductor topological transition by tuning a system parameter such as alloying composition or chemical doping^9^,^10^,^11^. For example, in zinc-blende crystals of HgCdTe the conical dispersion with some peculiar properties similar to those in Dirac and Weyl semimetals is achieved by changing cadmium concentration^11^. By varying a chemical composition and thereby tuning both the strength of spin-orbit coupling and the lattice parameters, one can also convert a system into a topological insulator^9^. Another possibility of tuning the topological order is to apply an external strain what was theoretically demonstrated for a number of narrow gap cubic semiconductors like *α*-Sn (grey tin), HgTe, and InSb^12^,^13^,^14^,^15^,^16^,^17^ or to combine the strain with adjusting the alloy composition^18^.

Up to now there is a lack of data concerning the electronic properties of ternary alkali bismuthides, such as KNa_2_Bi. Unlike the well known K_3_Bi and Na_3_Bi which have a hexagonal low temperature phase^19^,^20^ the crystal structure of KNa_2_Bi is cubic, 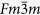
21. In this paper we theoretically demonstrate that KNa_2_Bi, a zero-gap semimetal with an inverted band structure at the Brillouin zone center, can be driven into different topological phases by applying pressure. We show that under hydrostatic compression the compound undergoes a transition to a conventional semiconductor with a conical Dirac-like dispersion of electronic bands at the point of the topological transition whereas upon uniaxial strain the compound can be converted into a topological insulator or into a three-dimensional Dirac semimetal with nontrivial Fermi arcs on the surfaces.

## Results and Discussion

KNa_2_Bi crystallizes in the cubic 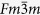 structure^21^ composed of four face-centered sublattices mutually shifted by 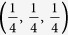 along the body diagonal. The sublattices occupied by Na atoms are symmetry equivalent. The lattice parameter, *a* = 7.97 Å, which is used in the calculation, was obtained by the total energy minimization and is slightly larger than the experimental value, *a*^*exp*^ = 7.896 Å. Positions of atoms are shown in Table 1.

### Equilibrium electronic structure

**Figure 1:**
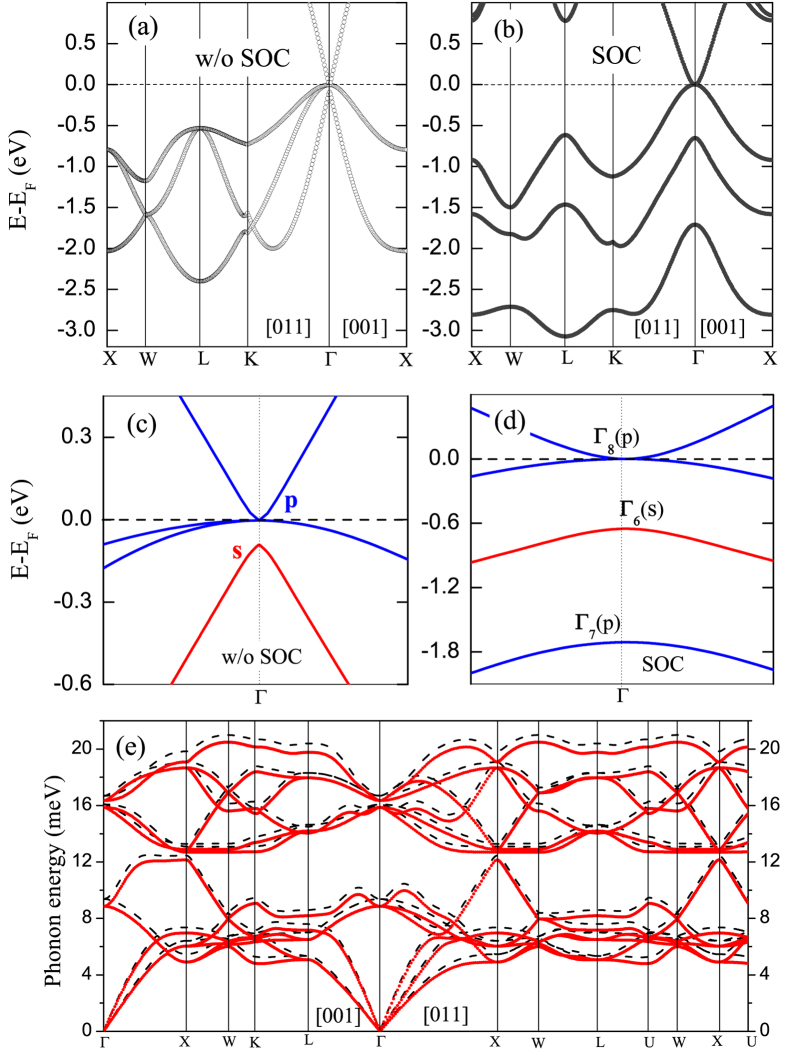
Electronic structure of KNa_2_Bi calculated (**a**) without spin-orbit coupling and (**b**) with spin-orbit interaction. (**c**,**d**) Bulk band dispersion in the vicinity of Γ shown to illustrate the effect of SOC and the band inversion. The bands consisted of *s*-like and *p*-like states in the Γ point are shown by red and blue lines, respectively. (**e**) Phonon spectrum of KNa_2_Bi calculated with (red solid lines) and without (black dashed lines) SOC.

Figure 1(a,b) displays the effect of spin-orbit coupling on electronic bands of KNa_2_Bi. We also calculated the phonon spectrum to show the dynamical stability of KNa_2_Bi in the cubic structure (Fig. 1(e)). It is obvious that low-energy electronic properties of KNa_2_Bi are determined by the bands located around the Γ point since away from the Brillouin zone center the valence and conduction bands are well separated. The arrangement of bands around the Fermi level near the BZ center is shown in Fig. 1(c,d).

In the absence of spin-orbit coupling (SOC), three bands which are linear combinations of *p*-like states are degenerate in the Γ point at the Fermi level, and the *s*-type band is lower in energy (Fig. 1(c)). The SOC changes the structure of the valence bands (Fig. 1(d)): three degenerate bands split into two Γ_8_ bands degenerate at Γ and a split-off state, Γ_7_. The *s*-type band, Γ_6_, is now between the Γ_8_ and Γ_7_ bands. Thus, with and without taking into account the spin-orbit interaction KNa_2_Bi is a zero-gap semimetal in which the conduction and valence bands are degenerate at the Γ point. The account of SOC cannot lift the degeneracy of the Γ_8_ bands, which is a consequence of the cubic symmetry.

Another feature is the inversion of band structure around the Γ point. According to the normal band-filling order, the valence band edge should be formed of Bi *p* orbitals while the conduction band edge should be composed of *s*-type states. However in KNa_2_Bi the Γ_6_ band appears below the Γ_8_ bands composed of Bi *p* states. The energy gap (*ε*^*S*^(Γ_6_) − *ε*^*P*^(Γ_8_)) is negative: about −0.09 eV in the calculation without spin-orbit coupling and increases up to −0.65 eV with the account of SOC.

### Electronic structure under hydrostatic lattice compression

**Figure 2:**
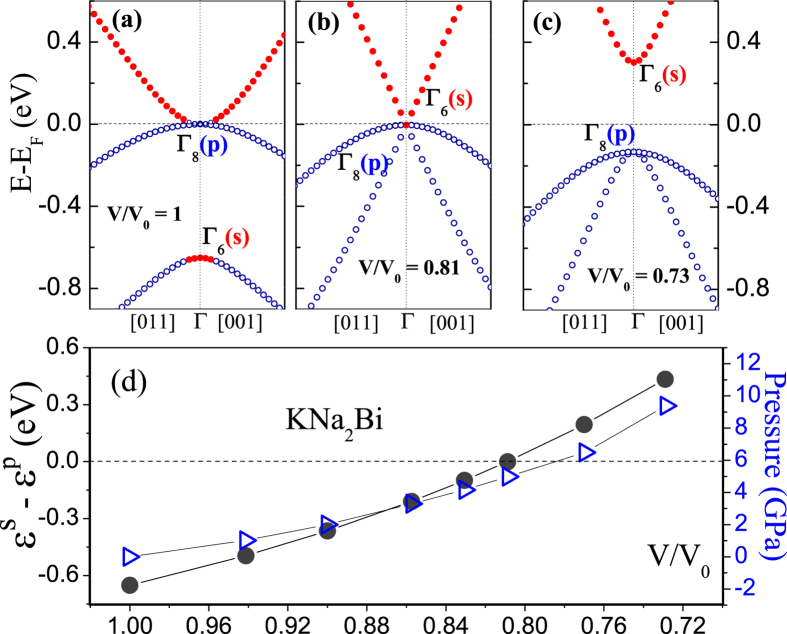
Evolution of the KNa_2_Bi electronic spectrum under hydrostatic lattice compression: (**a**) at *V*_0_ (*P* = 0, a topologically nontrivial semimetal), (**b**) *V*/*V*_0_ = 0.81 (*P* ≈ 5 GPa, the point of topological transition), and (**c**) *V*/*V*_0_ = 0.73 (*P* ≈ 9.4 GPa, a trivial gapped semiconductor). Open (blue) circles indicate the bands formed by Bi *p* orbitals while the bands mainly composed of *s*-type states are shown by solid (red) circles. (**d**) Energy gap *ε*^*S*^(Γ_6_) − *ε*^*P*^(Γ_8_) at the Γ point as a function of volume. The positive gaps correspond to a topologically trivial phase when the band inversion disappears. Open triangles show pressure values as a function of *V*/*V*_0_.

First we analyzed the electronic structure of KNa_2_Bi under hydrostatic lattice compression. Figure 2 shows the evolution of electronic bands under pressure in the vicinity of the Γ point. Upon the compression, the cubic phase remains dynamically stable. At low pressures, the compound is still a zero-gap semimetal with an inverted band order. The compression leads to a decrease of the gap between bands Γ_6_ and Γ_8_ at the Γ point. The variation of the energy gap with pressure is shown in Fig. 2(d). At *V*/*V*_0_ ≈ 0.81, the gap vanishes and all three bands become degenerate at the Fermi level (Fig. 2(b)). At the crossing point, two of the bands have a conical dispersion, which is crossed in the middle by the third band. A subsequent increase of pressure opens up an energy gap between the valence and conduction bands. Simultaneously the inverted band character around the Γ point disappears, i.e. the sequence of bands restores to normal: the *s*-type band, Γ_6_, is now above the Γ_8_ bands composed of Bi *p*-type states (Fig. 2(c)).

A similar semiconductor-to-semimetal topological transition in the crystals of HgCdTe was reported^11^. Upon tuning of the alloying composition the semiconductor band structure becomes gapless at the critical value of cadmium concentration and the conical band dispersion with peculiar properties appears at the BZ center.

### Electronic structure under uniaxial strain

**Figure 3:**
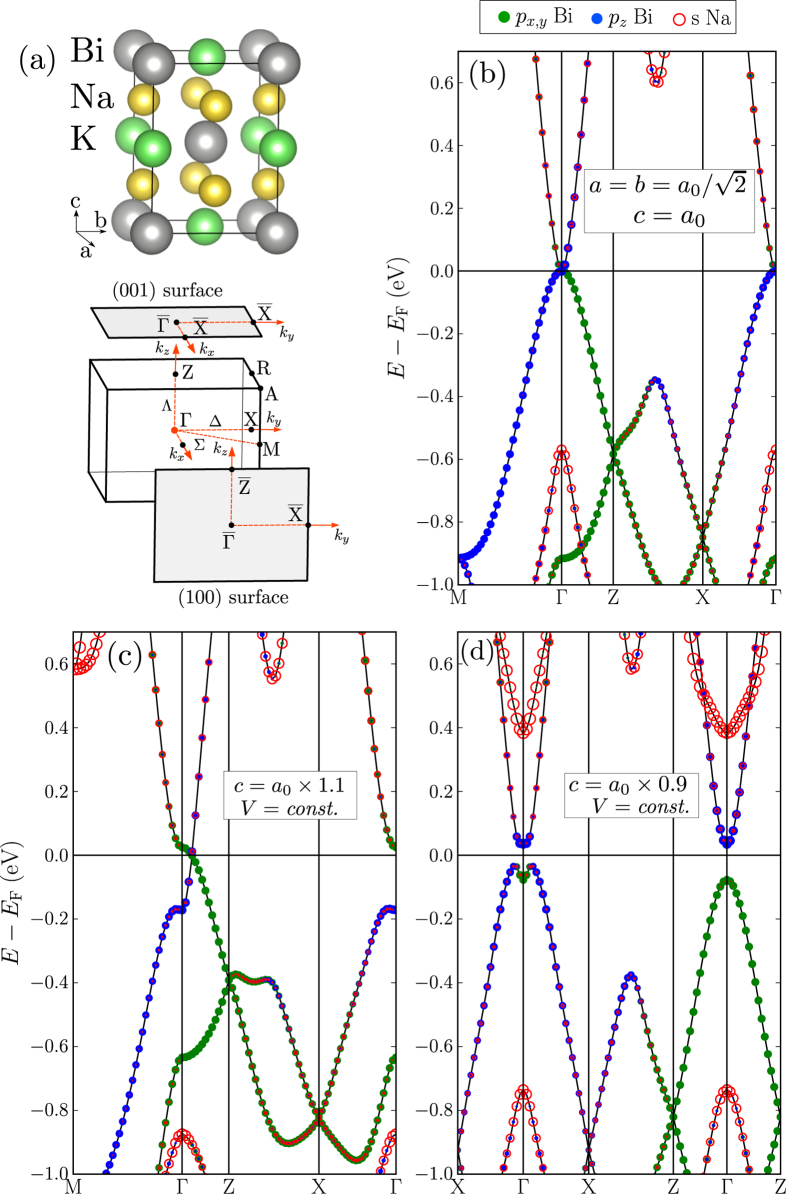
(**a**) (Top) Atomic positions in a tetragonal lattice and (bottom) high-symmetry points in the corresponding bulk Brillouin zone and in the projected surface BZ along the [001] and [100] directions. (**b**–**d**) Electronic band structure of KNa_2_Bi calculated (**b**) without strain and (**c**,**d**) under uniaxial lattice expansion/compression at constant volume.

Topological properties of KNa_2_Bi can be also changed by applying an external strain and thereby breaking the cubic symmetry. A strain was applied in the [001] direction (*c* axis). Such a strain lowers the crystal symmetry to a tetragonal one (Fig. 3(a)). The tetragonal structure of KNa_2_Bi is dynamically stable at all considered uniaxial strains. It turned out that the system responds differently to the compression and expansion: upon expansion the material becomes a 3D topological Dirac semimetal while the uniaxial compression converts the compound into a topological insulator. In both cases, the inverted band order in the vicinity of the Γ point remains that is an important sign of nontrivial topology. Figure 3(c,d) show the electronic band structure of KNa_2_Bi calculated with *c* = *a*_0_ ± 10% *a*_0_ at constant volume. The lattice optimization does not introduce any qualitative changes. To facilitate comparison we also used the primitive tetragonal unit cell to calculate the electronic structure of the unstrained compound (Fig. 3(b)).

#### A topological insulator

When a uniaxial compression breaks the cubic symmetry the degeneracy of the Γ_8_ bands is lifted. The splitting creates a direct energy gap at the Γ point between the valence *p*_*x*,*y*_-type Bi states and the conduction band composed mainly of *p*_*z*_ Bi orbitals (Fig. 3(d)). And an indirect energy gap appears near the Γ point because of the band inversion between Bi *p*_*x*,*y*_ and *p*_*z*_ states in the (*k*_*x*_, *k*_*y*_) plane. Since the inverted band order at the Γ point is preserved, the phase remains topologically nontrivial.

**Figure 4:**
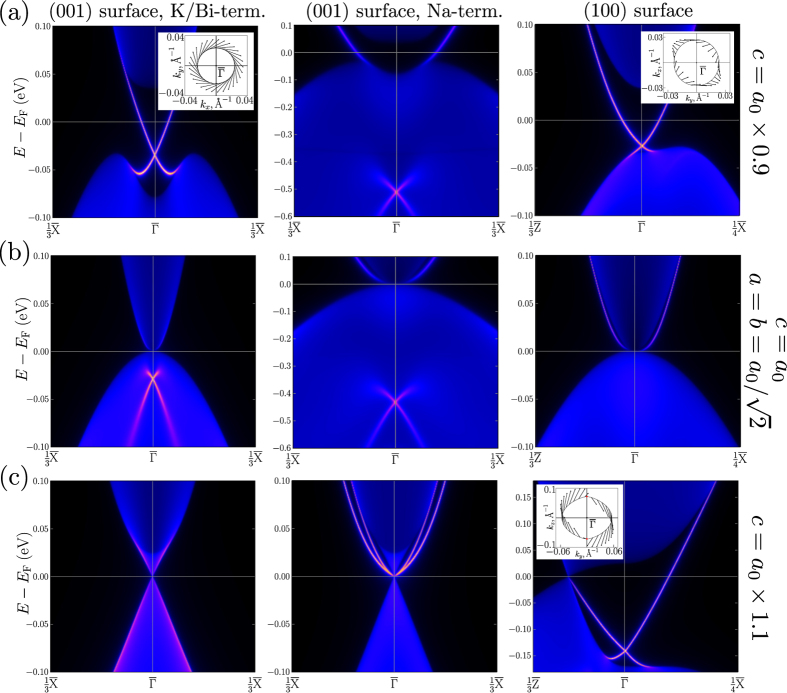
Projected densities of electronic states, obtained from *ab initio* based tight-binding model calculations for the semi-infinite systems: the Bi&K and Na terminated (001) surfaces and the (100) surface (**a**) under uniaxial lattice compression, (**b**) at the equilibrium lattice parameter, and (**c**) under uniaxial lattice expansion. The densities are shown by a color scale reflecting the localization in the outermost surface layer (in units of states/eV). Inserts show the corresponding Fermi surfaces with spin in-plane components for the topological surface states.

The topologically nontrivial character of the phase should be manifested in the existence of topological surface states. The calculated density of electronic states for the semi-infinite Bi&K- and Na-terminated (001) surfaces and for the (100) surface of KNa_2_Bi is shown in Fig. 4 together with the corresponding Fermi surfaces (in the inserts). All the data are presented as a color intensity plot. In the unstrained case (Fig. 4(b)), the surface states with a Dirac-type crossing are inside the projected valence bands due to the mixing with bulk electronic states. These topological surface states are clearly seen on the (001) surface (Fig. 4(b, left, middle)). The crossing (Dirac) point is located about 0.03 eV (0.45 eV) below the Fermi level depending on the surface termination.

Upon a uniaxial lattice compression, the surface Dirac point on the Bi&K-terminated (001) surface moves up towards the energy gap. With increasing strain, the surface states are split off from the gap edges and form a Dirac cone inside the gap (Fig. 4(a, left)). Such gapless topological states are also observed on surface (100) (Fig. 4(a, right)). On the (001) surface the spin texture in the upper part of the Dirac cone has a clockwise helical structure whereas in the case of the (100) surface it is strongly warped because of the lack of C_4_ symmetry in the (*k*_*z*_, *k*_*y*_) plane. Additionally, each layer along the [100] direction is composed of four crystallographically non equivalent atoms. In the case of Na termination, the topological states with a Dirac-type crossing remain inside the bulk valence bands (Fig. 4(a, middle)). However there is another surface state, it split off from the edges of the conduction bands, but when merging with bulk valence bands the state loses its surface character.

#### A Dirac semimetal

When a uniaxial expansion removes the band degeneracy at the Γ point a fundamental band gap does not appear (Fig. 3(c)). The valence and conduction bands cross at two points exactly at the Fermi level. As a result, a pair of 3D Dirac nodes shows up on the rotational *k*_*z*_ axis (the ΓZ direction) at ±*k*_*z*_ ≈ 0.2*π*/*c* (the position depends on applied strain). One of the crossing bands is composed of Bi 6*p*_*x*,*y*_ states and the other mostly of Bi 6*p*_*z*_ states. Since both bands are doubly degenerate each crossing point has a four-fold degeneracy.

The 3D Dirac cones and the inverted band order at the Γ point suggest the existence of nontrivial surface states (Fermi arcs). In the case of (001) surface, the projection of bulk electronic bands is superimposed on the nontrivial surface states arising owing to the band inversion at the Γ point and the Fermi surface represents just a point. The surface states show up along the edges of projected valence and conduction bands with the crossing at the Γ point (Fig. 4(c, left)).

On the (100) surface, however, the projected bulk Dirac nodes and the nontrivial surface states are separated in the momentum space and can be visible (Fig. 4(c, right)). The Fermi surface composed of two half-circle Fermi arcs is closed with two singular points corresponding to the projection of bulk Dirac nodes. The spin texture of Fermi arcs has a helical structure, but the value of spin is not defined at the singular points.

## Discussion

To describe the change in the band dispersion at the point of extremum (the Γ point) under pressure, we have constructed a low-energy effective **k** · **p**-model Hamiltonian^22^ by considering the rotational (C_4_, along the *k*_*z*_ axis), time-reversal and inversion symmetries. Since the inverted band order in the vicinity of Γ is a characteristic feature of the compound and is conserved under any uniaxial strain we do no consider *s*-type states. Only low-energy electronic states around the Γ point which are mostly consist of Bi 6*p*_*x*,*y*,*z*_ orbitals are used as basis states: 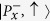, 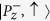, 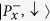 and 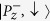. The superscript indicates the parity, and the arrow — the direction of spin moment. Due to the in-plane isotropy, components 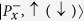 can be easily replaced by 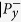 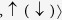. The coupling of the basis states with the same parity (p-orbital hybridization) is also accounted for. So the parity operator is *τ*_0_, a zero Pauly matrix. In such a basis set, the Hamiltonian can be written as


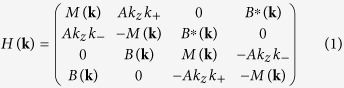


**Figure 5:**
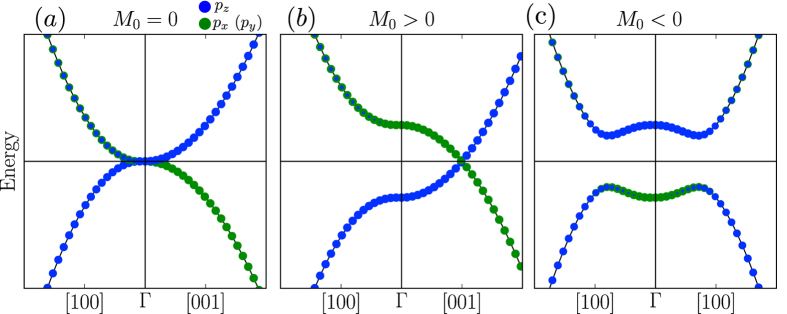
Band dispersions obtained from *H*(**k**) (eq. 1) with (**a**) *M* = 0, (**b**) *M* > 0 and (**c**) *M* < 0. The size of green (blue) circles reflects the contribution of 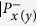 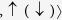 and 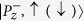 electronic states to the bands.

Here 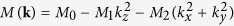, 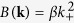, *k*_±_ = *k*_*x*_ ± *ik*_*y*_. So the parameters of the effective model are *M*_0,1,2_, *A* and *β*. In the Hamiltonian the topology of band structure is solely determined by parameter *M*_0_ which corresponds to the energy difference between basis orbitals. Other parameters are fixed in our calculation so as to reproduce both topological transitions: *M*_1_ = 1.0 eVÅ^2^, *M*_2_ = −1.0 eVÅ^2^, *A* = 1.0 eVÅ^2^, and *β* = 1.0 eVÅ). If *M*_0_*M*_1_ < 0 the system is a topological insulator, otherwise (*M*_0_*M*_1_ > 0) it is a Dirac semimetal. The spectra of Hamiltonian, 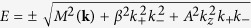, are presented in Fig. 5.

Let us consider three possible situation: *M*_0_ = 0, *M*_0_ > 0 and *M*_0_ < 0. If *M*_0_ = 0 (Fig. 5(a)) the compound is a zero-gap semimetal. Two double degenerate bands with square dispersion along all the directions of the BZ are touching at the Γ point and the crossing point has a four-fold degeneracy. Under hydrostatic compression, the stability of the system in the topological semimetal phase (*M* = 0) is provided by the cubic symmetry when all the basis orbitals are equivalent and have the same energy. To make *M*_0_ non-zero the band degeneracy at the Γ point should be lifted. Upon positive (negative) uniaxial strain, *E*(|*P*_*z*_, ↑(↓)〉) is greater (less) than *E*(|*P*_*x*(*y*)_, ↑(↓)〉) and, so, *M*_0_ < 0 (*M*_0_ > 0). In the case of *M*_0_ > 0 (Fig. 5(b)), the band gap is not global. The system posses a semimetal band structure with two Dirac points at 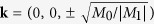. Along the *k*_*x*_ direction the gapped bands have a square dispersion. The case of *M*_0_ < 0 (Fig. 5(c)) corresponds to a topological insulator with hybridized gap. All three presented situations are similar to the tight-binding results.

An analysis of the parity of occupied electronic states also reveals two phases in KNa_2_Bi from the viewpoint of 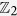 index classification. The 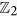 index was obtained from the parity of occupied electronic states at the time-reversal invariant points of the bulk Brillouin zone^12^ and also using Z2Pack software package. In the case of topological insulator (*M*_0_ < 0), the invariants are 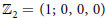. It coincides with the results of surface band structure calculations: topological surface states exist on all possible surfaces. In the case of a Dirac semimetal (*M*_0_ > 0), the situation is different: 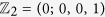 that corresponds to a weak topological insulating phase. This fact is also supported by the existence of surface states only on the (100) and (010) surfaces.

To describe possible spin textures of surface states let us consider the Hamiltonian consisting of the spin-orbit contribution, which includes both Bychkov-Rashba (BR) and Dresselhaus terms, and an additional “kinetic”-like term^23^:


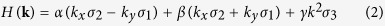


Here *k*_*x*_, *k*_*y*_ are in-plane components of momentum, 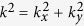. *α* and *β* determine the strength of the RB and Dresselhaus spin-orbit interactions and *γ* defines the “kinetic”-like term. *σ*_1_, *σ*_2_ and *σ*_3_ are Pauly matrices. The BR and “kinetic”-like terms are isotropic, the Dresselhaus term has C_2_ symmetry and so can be considered as a warping.

**Figure 6:**
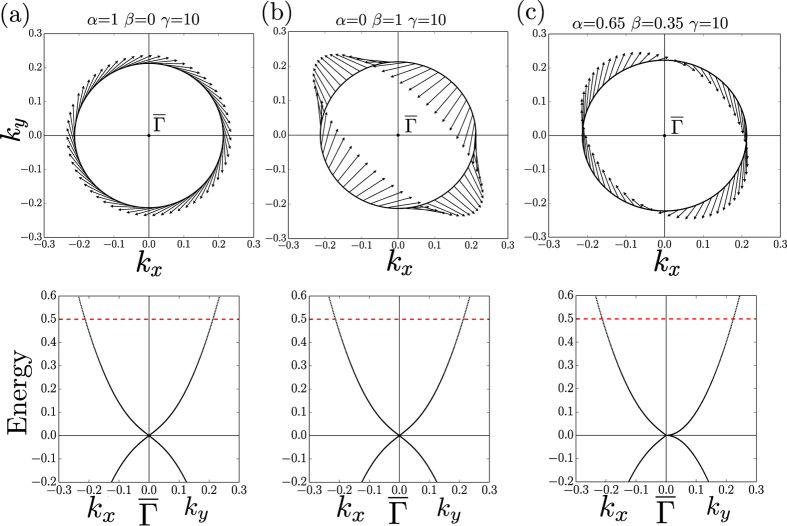
Spin texture of isoenergetic contours in the upper part of a Dirac cone on the basis of the proposed Hamiltonian (eq. 2). (**a**) *α* = 1, *β* = 0, *γ* = 10 (**b**) *α* = 0, *β* = 1, *γ* = 10 (**c**) *α* = 0.65, *β* = 0.35, *γ* = 10. The energy level is shown by a red dashed line.

In Fig. 6 three spin configurations corresponding to those shown in Fig. 4 are presented. Upon uniaxial compression, on the (001) surface of KNa_2_Bi (Fig. 4(a, left)) the spin texture in the upper part of the Dirac cone has a strong clockwise helicity without any warping. To obtain a similar spin texture we set *α* = 1, *β* = 0, *γ* = 10 (Fig. 6(a)). In this case, only the BR interaction contributes to the in-plane components of spin while the out-of-plane component is determined by the “kinetic”-like term. The existence of the non-zero out-of-plane spin component is proved by our TB calculations. The “kinetic” term also reduces the radius of isoenergetic contour and leads to a square dispersion of surface states with increasing **k**-vector.

The in-plane spin texture in the Dirac cone on the (100) surface (Fig. 4(a, left)) is fully described by the Dresselhaus term which has a C_2_ symmetry (we set *α* = 0, *β* = 1, *γ* = 10, Fig. 6(b)). In this case the spin texture is strongly warped because of the lack of C_4_ rotational symmetry on the (100) and (010) surfaces due to the strain along the [001] direction. Such a warping was also observed in the spin texture of bulk states in the tensile-strained HgTe^24^. In both considered cases there is no warping in the energy spectrum: the dispersions along *k*_*x*_ and *k*_*y*_ directions are the same.

The most interesting is the case of surface states on the (100) surface of KNa_2_Bi under uniaxial expansion (Fig. 4(c, right)). If in the previous cases the spin texture can be described using either the BR or Dresselhaus contributions, in this case, you need to consider all three terms in the Hamiltonian. The difference between *α* and *β* leads to spin helicity, so if *α* = *β*, the spin will be directed strictly along the positive (negative) *k*_*y*_ axis, depending on whether *k*_*x*_ < 0 (*k*_*x*_ > 0). Finally, the “kinetic”-like term results in a more isotropic dispersion. Without this term (*γ* = 0) in the *α* = *β* limit the isoenergetic contour would consist of two parallel lines with the opposite spin direction. If *α* ≠ *β* the dispersion along the *k*_*x*_ and *k*_*y*_ directions is different (Fig. 6(c)). And when *k*_*x*_ = 0 the in-plane components of spin disappear, which corresponds to the bulk Dirac points. Thus, using a simple **k** · **p**-model, we can describe all possible spin texture configurations on the surfaces of KNa_2_Bi. This model can also be used for related compounds with cubic symmetry.

Similar strain-induced transitions were predicted theoretically for gray tin (*α*-Sn) and HgTe^12^,^13^,^16^,^17^,^25^. The existence of topological surface states in the strained HgTe was also demonstrated experimentally by ARPES measurements^14^. At zero pressure, like KNa_2_Bi, *α*-Sn and HgTe are gapless semimetals with the inverted bulk band structure in the vicinity of Γ and surface states located on the edge of the conduction bands and inside the valence bands, indicating a nontrivial topology^25^. These semimetals are turned into 3D topological insulators by applying a uniaxial compression^12^. The strain induces a band gap in the bulk whereas the topological surface states acquire a gapless Dirac-cone dispersion, thereby giving a metallic character to the 3D topological insulators^12^,^13^,^25^. Like in the case of KNa_2_Bi the gapless surface states in HgTe is found to be sensitive to the surface termination^14^. Under uniaxial expansion of HgTe the degeneracy at Γ is also lifted but a fundamental band gap does not appear. Like in KNa_2_Bi the valence and conduction bands form a pair of Dirac points in the Z–Γ–Z line^18^.

## Conclusion

We have analyzed theoretically the effect of external strain on the electronic properties of KNa_2_Bi, a cubic bialkali bismuthide. Under hydrostatic compression KNa_2_Bi, a zero-gap semimetal with the inverted band order around the BZ center, undergoes a transition to a conventional gapped semiconductor with a conical Dirac-like dispersion of electronic bands at the point of the topological transition. Simultaneously the sequence of different symmetry bands restores to normal. We find that topological Dirac-like surface states exist even in the unstrained compound as long as the band structure is inverted. It is also shown that KNa_2_Bi can be driven into distinct topologically nontrivial phases by breaking the cubic symmetry. Under uniaxial lattice expansion the compound is converted into a three-dimensional Dirac semimetal with nontrivial Fermi arcs on the surface while a nontrivial topological insulating phase can be realized by applying a uniaxial lattice compression.

## Methods

The electronic structure calculations were performed in the mixed-basis pseudopotential approach^26^,^27^,^28^ with the exchange and correlation energy functional evaluated within the generalized gradient approximation^29^. Spin-orbit coupling (SOC) was incorporated into the pseudopotential scheme via Kleinman’s formulation^30^ and was treated fully self-consistently^31^. Phonon dispersions were calculated using the linear response technique^32^ in combination with the mixed-basis pseudopotential method^28^. Integrations over the Brillouin zone (BZ) were performed by sampling a 8 × 8 × 8 mesh corresponding to 29 and 75 *k* points in the irreducible part of the cubic and tetragonal BZ, respectively, combined with a Gaussian broadening with a smearing parameter of 0.01 eV. The projected density of surface states was calculated using *ab initio* based tight-binding formalism^33^,^34^.

## Additional Information

**How to cite this article**: Sklyadneva, I. Y. *et al*. Pressure-induced topological phases of KNa_2_Bi. *Sci. Rep*. **6**, 24137; doi: 10.1038/srep24137 (2016).

## Figures and Tables

**Table 1:** Positions of atoms in the cubic 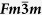 structure.

Position	KNa_2_Bi		*a*	K - Bi	Na - Bi
(1/2, 1/2, 1/2)	K	theory	7.97	3.98	3.45
±(1/4, 1/4, 1/4)	Na	expt.^21^	7.896	3.95	3.42
(0, 0, 0)	Bi				

Lattice parameters (*a*) and interatomic distances between Bi and alkaline atoms are given in Å.
